# Gratitude, resignation and the desire for dignity: lived experience of food charity recipients and their recommendations for improvement, Perth, Western Australia

**DOI:** 10.1017/S1368980018001428

**Published:** 2018-06-27

**Authors:** Sue Booth, Andrea Begley, Bruce Mackintosh, Deborah Anne Kerr, Jonine Jancey, Martin Caraher, Jill Whelan, Christina Mary Pollard

**Affiliations:** 1 College of Medicine & Public Health, Flinders University, GPO Box 2100, Adelaide, SA 5000, Australia; 2 Faculty of Health Science, School of Public Health, Curtin University, Perth, WA, Australia; 3 School of Agriculture and Environment, The University of Western Australia, Perth, WA, Australia; 4 Centre for Food Policy, City University of London, London, UK; 5 Faculty of Health, School of Health Science and Social Development, Deakin University, Geelong, VIC, Australia; 6 Public Health Division, Department of Health, Government of Western Australia, Perth, WA, Australia

**Keywords:** Charitable food system, Charitable food services, Food charity, Recipient perspective, Food insecurity, Nutrition, Voluntary failure

## Abstract

**Objective:**

The present study explored recipients’ perceptions of food charity and their suggested improvements in inner-city Perth, Western Australia.

**Design:**

In-depth interviews were conducted with charitable food service (CFS) recipients. Transcripts were thematically analysed using a phenomenological approach.

**Setting:**

Interviews were conducted at two CFS in inner-city Perth.

**Subjects:**

Fourteen adults.

**Results:**

The recipients’ journeys to a reliance on CFS were varied and multifactorial, with poverty, medical issues and homelessness common. The length of time recipients had relied on food charity ranged from 8 months to over 40 years. Most were ‘grateful yet resigned’, appreciative of any food and resigned to the poor quality, monotony and their unmet individual preferences. They wanted healthier food, more variety and better quality. Accessing services was described as a ‘full-time job’ fraught with unreliable information and transport difficulties. They called for improved information and assistance with transport. ‘Eroded dignity’ resulted from being fed without any choice and queuing for food in public places, often in a volatile environment. ‘Food memories and inclusion’ reflected a desire for commensality. Recipients suggested services offer choice and promote independence, focusing on their needs both physical and social.

**Conclusions:**

Although grateful, long-term CFS recipients described what constitutes a voluntary failure. Their service improvement recommendations can help meet their nutritional and social needs. A successful CFS provides a food service that prioritises nutritious, good-quality food and individual need, while promoting dignity and social inclusion, challenging in the current Australian context.

Compared with other nations with entrenched food insecurity, Australia is still coming to grips with this issue that is increasing due to unemployment, the economic downturn and a contracting welfare safety net^(^ ^1^ ^)^. In contrast to countries with government-funded food relief programmes^(^ ^2^ ^)^ or regulations diverting food waste to charities^(^ ^3^ ^,^ ^4^ ^)^, Australian food insecurity responses are *ad hoc.* Food charity, the delivery of donated, unsaleable or waste food by the voluntary (non-profit) sector to those in need, is the dominant Australian response^(^ ^5^ ^)^. Originally designed to provide immediate short-term food relief, the system is struggling as food insecurity and the demand for food assistance are chronic and increasing^(^ ^5^ ^,^ ^6^ ^)^. Although charitable food services (CFS) alleviate short-term hunger, they are not equipped to address persistent and regular requests for food or support the nutritional needs of those with chronic health issues such as diabetes or HIV/AIDS^(^ ^7^ ^,^ ^8^ ^)^.

The non-profit sector’s ability to effectively address problems such as food insecurity has been questioned, particularly when it is reliant predominantly on philanthropy^(^ ^6^ ^,^ ^9^ ^)^. In fact, Salamon’s Theory of Voluntary Failure was developed to explain the effectiveness of the voluntary response to issues such as food insecurity^(^ ^10^ ^)^. The expression ‘voluntary [sector] failure’ refers to ‘situations in which non-profits cannot adequately provide a service or address a social problem at a scale necessary for it’s alleviation’^(^ ^11^ ^)^ (p. 119). The voluntary sector may fail for four reasons: (i) philanthropic insufficiency, which refers to inadequate resources; (ii) philanthropic particularism, which is a focus on a specific subgroup of the poor; (iii) philanthropic paternalism, which occurs when voluntary sector staff work with clients in ways that undermine clients’ independence and dignity; and (iv) philanthropic amateurism, which refers to the lack of training and professionalism in the non-profit sector. Philanthropic insufficiency, where non-profits lack sufficient resources to meet demand, appears to be occurring in Australia. Between 2015 and 2016, emergency relief agencies reported an 8 % increase in people requiring food assistance^(^ ^12^ ^,^ ^13^ ^)^. Of particular concern are the 43000 people per month unable to be assisted, a third of whom are children^(^ ^13^ ^)^.

In Western Australia, food charity is provided by a diverse range of non-profit organisations with different operating models, funding and underpinning philosophies. Within the sector, services can be described as ‘direct’ or ‘indirect’. ‘Direct’ services provide food charity directly to people in need (i.e. a face-to-face interaction, such as food vans and parcels). ‘Indirect’ services (e.g. food banking and rescue organisations) provide food to ‘direct’ services who distribute it to those in need. In short, ‘indirect’ services operate at arm’s length from clients. Food is distributed via food pantries, mobile vans, food parcels and supermarket vouchers. In 2015, seventeen direct-service organisations provided food charity in inner-city Perth, the capital city of Western Australia.

Evidence from the USA and Canada indicates some of the food provided by CFS is nutritionally suboptimal^(^ ^14^ ^–^ ^17^ ^)^. A systematic review of the nutritional quality of food provided by food pantries found low amounts of milk products, vitamins A and C and calcium^(^ ^14^ ^)^. Little Australian information on the nutritional adequacy of CFS meals exists. Compared with domiciled young people, the nutrient intake of 150 homeless young people reliant on CFS in Adelaide was inadequate, lacked variety, and contained less fruit, vegetables, bread and cereal servings^(^ ^18^ ^)^.

Notably absent from the discourse on food charity are the voices of CFS recipients. Including the voices of disempowered people provides an understanding of their experience essential for co-designing interventions^(^ ^19^ ^)^. Studies that place the experiences, needs and voices of people experiencing food insecurity at the forefront are critical^(^ ^20^ ^)^. Important questions include: what is it like to receive food charity? Is the quality and quantity of the food provided adequate? The current paper presents recipients’ perspectives on CFS located in inner-city Perth and their suggestions for improvement.

## Methodology

The present research is part of a mixed-methods study to define the scope and nature of CFS required to achieve adequate nutrition. The current component uses a phenomenological approach^(^ ^21^ ^)^ to understand the lived experience or the phenomenon of relying on food charity and recommendations for improvement. The study was conducted according to guidelines in the Declaration of Helsinki and all procedures involving human subjects were approved by the Curtin University Human Research Ethics Committee (HR183/2015). Written informed consent was obtained from all subjects; however, in cases where respondents could not read, verbal informed consent was witnessed and formally recorded.

### Instrument

**Fig. 1:**
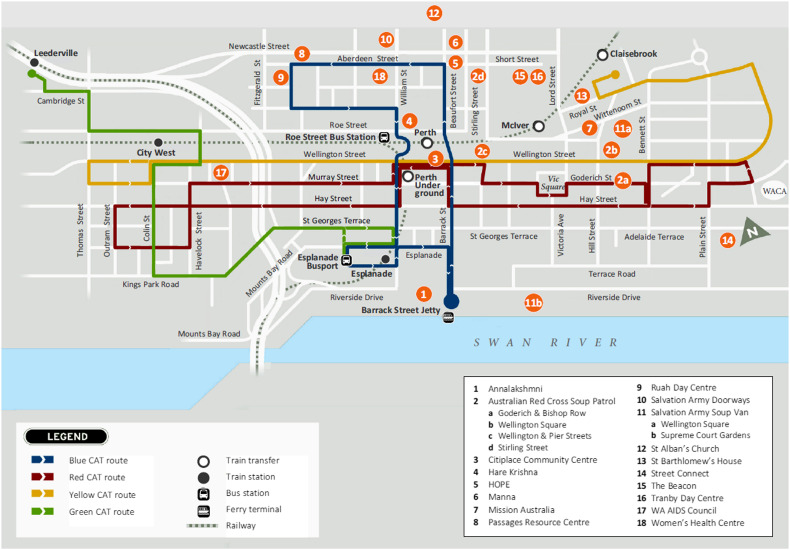
Map of charitable food services in inner-city Perth, Western Australia, after disclosure of service locations used by interviewees, February 2016

A semi-structured interview schedule was developed based on previous research and the authors’ experience to explore the context of CFS usage, food procurement, appropriateness of services and suggestions for improvement. After piloting, minor adjustments were made to the phrasing and order of questions. Box 1 shows the interview schedule. Figure 1 shows the updated map of CFS and transport routes that were used during interviews as a visual cue to prompt recipients^(^ ^22^ ^)^.

Box 1 Semi-structured interview schedule

**Table:** 

Participant’s: ∙ Usual pattern of accessing food from charitable food services. The ‘what, where, how and why’ of accessing charitable food in the city. ∙ Perceptions of the appropriateness of charitable food services. ∙ Barriers and enablers to accessing charitable food services. ∙ Perceptions of effectiveness/how well charitable food services are currently meeting their needs and the needs of people more broadly. ∙ Views on the challenges faced by people who rely on charitable food services. ∙ Practical suggestions for charitable food service improvement. ∙ Feedback for agencies involved in charitable food services.

### Participants

The research assistant (J.P.), a CFS volunteer, undertook recruitment. A convenience sample was drawn from adults attending two CFS, a drop-in centre and a multipurpose humanitarian service. The study aims and procedures were explained to potential respondents and an information sheet provided. Confidential interviews were conducted in an office within the service and recorded with consent. Limited demographics were collected, in order to build rapport and foster trust. Interviews lasted 15–60 min after which recipients received $AU 10 cash in recognition of their time. Interviews continued until no new information was elicited. There were no refusals and fifteen interviews were conducted with one unusable due to incomprehensible responses.

### Analysis

Field notes were written up within 24 h using pseudonyms. Recordings were professionally transcribed verbatim and all recipients declined to review their transcripts.

A four-stage thematic analysis^(^ ^23^ ^)^ was undertaken, commencing with stage 1: data immersion. The interviewer (S.B.) read and re-read the interviews and a sample (*n* 7) was checked against the recordings for accuracy. In stage 2, all interview transcripts were entered into NVivo version 10.10.2 qualitative software for data management. Interviews were coded using an inductive approach. In stage 3, coded data were organised into nodes and then reviewed (by C.M.P.). Then, S.B. and C.M.P. independently summarised codes into key themes which were then compared and discussed, resulting in an agreed set of themes (stage 4).

## Results

Fourteen interviews were included in the analysis, see Table 1 for participant demographics. The length of time using CFS ranged from 8 months to over 40 years. Seven recipients used CFS for 20 years or more and two described intergenerational use. Poverty, illness and homelessness were commonly reported circumstances leading recipients to food charity. Escalating costs of living and housing were the main reasons cited by eight interviewees. Five recipients relied on food charity because they were homeless and five mentioned a mental health diagnosis or hospitalisation.

**Table 1:** Summary of interviewee demographics: charitable food service (CFS) recipients (*n* 14) in inner-city Perth, Western Australia, February 2016

Pseudonym	Gender	Age (years)*	Stated length of time using CFS
Barbara	F	~mid 30s	Last 8 months
Rick	M	~early 40s	~8 months
Andrew	M	50	10 months
Debbie	F	59	Consistent monthly use over last 12 months
Jane	F	45	16–20 months
Bob	M	~mid–late 40s	3·5 years
Joe	M	32	4–5 years
Sandy	F	74	5 years
Nathan	M	~mid–late 40s	5 years
Clint	M	~early–mid 30s	5·5 years
Chris	M	~late 40s–early 50s	12 years
Lynda	F	37	22 years
Rachel	F	63	Approx. 30 years
David	M	47	Approx. 40+ years

* Age estimated (indicated by ~) or as stated by participant.

### Key themes

**Fig. 2:**
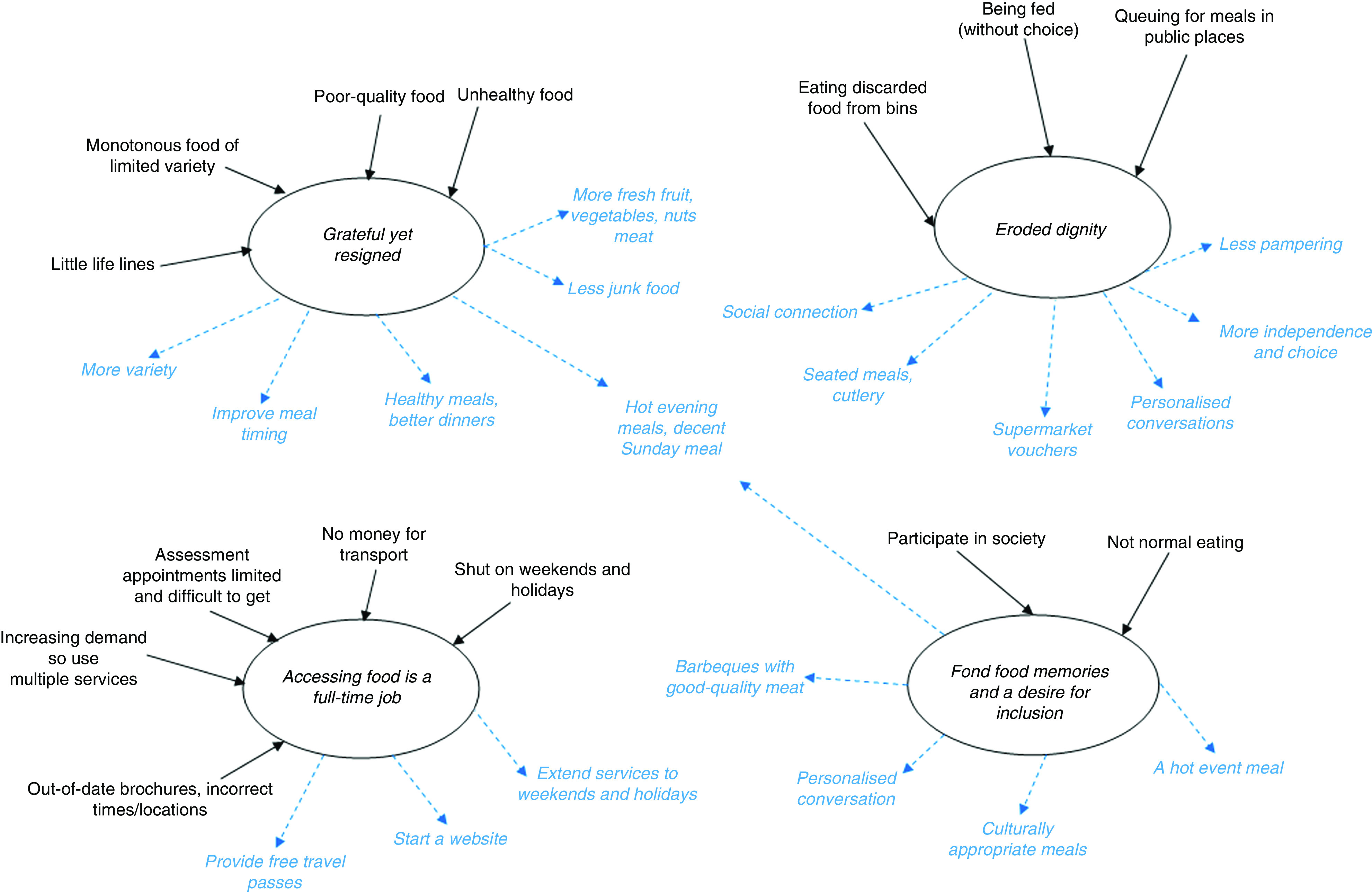
(colour online) Key themes (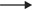, emergent themes) and recommendations (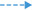) for improvements derived from interviews with charitable food service recipients (*n* 14) in inner-city Perth, Western Australia, February 2016

Four themes emerged: (i) ‘grateful yet resigned’; (ii) ‘a full-time job’; (iii) ‘eroded dignity’; and (iv) ‘food memories and inclusion’. The themes and interviewees’ recommendations for improvement are presented below and shown in Fig. 2.

#### Grateful yet resigned

Recipients were ‘grateful yet resigned’, appreciative of ‘any’ food and resigned to the poor quality, monotony and their unmet individual food preferences.

There was a reluctance to criticise CFS and six recipients expressed gratitude and appreciation for the food provided and for the non-judgemental attitudes of staff: ‘Thank God, I don’t know what I’d do without them’ and calling them ‘little lifelines’. When asked directly, nine recipients said the CFS met their immediate needs and only two said the food did not entirely meet their needs:

‘No, … they’re too greasy and no, they don’t get any fresh meat in their meals.’ (Lynda)

There was acceptance and humour regarding the food and the phrase ‘beggars can’t be choosers’ prevailed, probably because the food was free and they were hungry:

‘So … as far as the food goes, the food quality is fine… Sometimes the pasta is like eating rubber, it’s overcooked but you know you can’t ask for “al dente” in a place that hands out free food.’ (Andrew)

Considerable commentary indicated that CFS provide basic, low-quality food to satiate immediate hunger. Most food was described as monotonous, packaged and lacking in nutrients, for example:

‘No, it’s never changed, it’s just the same ... I reckon they should do something, why not just put out different meals or something ... for the people to select whatever they want.’ (Lynda)

For domiciled recipients, food charity often consisted of ready-made parcels including canned or packaged staple foods such as baked beans, breakfast cereal and soups, described as ‘survival’ or ‘cupboard’ food. They described the food as easy to heat up, uncomplicated and ‘it fills you up’, but it ‘hasn’t got all the vitamins in it’. Seven long-term CFS recipients talked about food quality and chefs. Hostel dwellers with access to centralised food services spoke of precooked meals, repetition, recycling of leftovers, limited choice and inability to accommodate individual preferences.

When directly asked if the CFS food provided was healthy, responses were mixed. There was some access to fresh fruit and vegetables, but increased access was desired. Cheap, fatty meat in sausages and meat pies provided by some CFS were viewed with disdain. Recipients observed that unhealthy packaged foods included in parcels, such as potato crisps or sugary foods, helped them to ‘feel good’ and provided comfort during difficult times. Long-term recipients lamented the lack of choice in food parcels and the proliferation of unhealthy processed foods:

‘It never used to be like that … they used to ask me you know, braised steak and onions, camp pie, three choices, and I’d just say camp pie … They didn’t even do that … they just gave me things packed up and there was a lot of junk food … I was disappointed.’ (Rachel)

Recipients acknowledged that personal food preferences and dietary requirements are difficult to accommodate. They recommended expanding food variety to allow people to choose the food they want to eat. Healthy food was recommended by five recipients, particularly those trying to manage chronic conditions. Jane, a diabetic, noted the compromises required when CFS provide predominantly unhealthy food. She recommended reducing the amount of snack food provided, as did Andrew:

‘They do a lot of soups … that are quite heavy creamy-based so I don’t really eat a lot … I am not going to complain too much, but more fresh fruit and less of the sugary stuff would be good.’ (Andrew)

Recipients described a plethora of CFS resulting in bountiful food provision, albeit without much choice and often of poor quality. Six recipients described the overabundance of food as ‘like the garden of Eden’ or ‘being rich without being rich’. Some described eating multiple daily breakfasts sourced from different services. However, as one male recipient pointed out, ‘there’s always plenty, [of food] ... quantity it’s not the issue, but it’s more the quality’. Lynda explained the paradox of abundance and restrictions, with desired foods sometimes in limited quantity:

‘They give out extra sandwiches and that, they can’t give extra pies, only if they’ve got enough … some men and pregnant women would like to get extra but can’t.’ (Lynda)

Most regularly accessed enough food from CFS and added variety via CFS-issued supermarket vouchers or purchased food with their own money. Purchasing food allowed choice, independence and participation in mainstream life. Some were highly skilled in their ability to purchase food for two weeks using a $AU 20 voucher:

‘They gave me a $20 voucher for [supermarket], so I used to go buy the twelve packs of hot dog rolls and then I get … turkey meat or chicken … I had that pre-cut for $11, and then cheese and dip, just for a little bit of spread, and that lasted me nearly two weeks.’ (Clint)

Food quality in residential short-term accommodation was also questionable. A perceived skill deficit among chefs was common knowledge:

‘Sometimes at the [hostel] they serve you lunch because it’s precooked or you go up and help yourself, depends what they’ve cooked … sometimes they use leftovers … so make the lunches, like sandwiches … Two of the chefs there they can’t cook for crap.’ (Rick)

Recipients also wanted improved meal timing and appropriate temperatures, including ‘hot evening meals’. Suggestions were made to change the eating experience and types of food (with a preference and desire for healthy food). Specifically, to include ‘better dinners’ with ‘more variety’ and ‘more fresh fruit, vegetables, nuts and good-quality meat’ and ‘less sugary, treaty chocolatey foods and creamy soups or junk food’ and the ability to choose foods to assist chronic disease management.

### A full-time job

Accessing information on the CFS system was described as ‘a full-time job’. Recipients reported difficulties obtaining accurate information about where, when, what was available and the eligibility criteria for food assistance. Assessment appointments were offered only on certain days and finding out how to get an appointment was challenging. Brochures were often out of date and incorrect:

‘There is a few brochures that do get handed out, but I found a lot of them out of date, wrong times, wrong days, some [services] don’t even exist anymore. It’s a huge process of elimination of what works and what doesn’t … so it’s basically a process of word of mouth.’ (Barbara)

Long-term recipients described a pattern of CFS usage and sharing information or sometimes accompanying newcomers and showing them how to navigate free transport. Despite being knowledgeable, several recipients asked researchers for a copy of the interview prompt map.

Accessing CFS during weekends, public holidays and major holiday periods was difficult, particularly between Christmas and New Year. Interviewees spoke of receiving a Christmas food hamper and how they stockpiled food and had to forward plan to ‘get through’ until services resumed:

‘But if it’s the Christmas period they usually lock down for two weeks. So over those two weeks you’ve got to make sure, we got hampers.’ (Nathan)

For some, getting food was resource-intensive because they did not like asking for help and used the CFS only intermittently. They acknowledged the growing demand for food and perceived that CFS were under pressure. Consequently, they were conservative in their service usage and tried to avoid relying on any one specific service:

‘I know they’ve got a lot of people to look after, so I basically try and go to other places as well … the funding with some charities makes it pretty hard.’ (Barbara)

Food access challenges were also reflected in the multiple ways food was sourced. Eight purchased food, seven used friends or neighbours, five relied on family and one had begged. One interviewee disclosed that on several occasions she had eaten discarded food such as half-eaten lunches found in bins, and she had acquired plastic utensils for future opportunities.

Ongoing work was required to source sufficient food from different CFS to meet needs. Most recipients demonstrated an excellent, almost encyclopaedic knowledge of the city, recall of free bus timetables and the routes to reach CFS. Sandy walked two hours to access CFS or took the 5 a.m. bus because it’s free for pensioners before 6 a.m. Arriving at 5:30 a.m. on the day of the interview, she slept on a park bench until 9 a.m. when the service opened.

Allowing sufficient walking time to arrive at mobile CFS and not miss out on food was a consideration. Seven recipients occasionally used public transport, which was stressful as they could not afford the $AU 1.80 fare. They ‘jumped the train’ without a valid ticket and had to be hyper-vigilant to avoid the $AU 200 fare aversion fine.

Recipients wanted better coordination of CFS activities to meet their needs. The recipients’ experience was somewhat fractured with CFS seemingly operating independently with substantial service gaps such as no evening meals on weekends. A website containing basic service information with recipient feedback opportunities was requested:

‘I would love for them to start a website … to put all the information into one spot, where you could have all the facilities that are available in the area, what they provide, what times you call them … getting back to the information and standardising stuff. I’d love to do a website and get feedback from people on the street, what organisations and what things actually do work.’ (Barbara)

Interviewees recommended extended opening hours on evenings, weekends and public holidays and free travel passes to facilitate better access.

### Eroded dignity

Dignity eroded as a result of being fed without choice and queuing for food in public places, often in a potentially threatening environment. Food queues were spoken about extensively, and were seen as necessary but frustrating, undignified, highly visible and sometimes leading to conflict. Jostling for queue position was a recognised conflict trigger. Long queues meant long waits, undesirable especially in extreme weather. Fear of missing out on food was exacerbated with long queues at mobile van services. Long-term recipients noted a shift in the queue demographics, describing backpackers and pensioners as easily spotted ‘newcomers’. Newcomers were a source of friction, fuelling the perception that ‘regulars’ might miss out on food:

‘I get into line with people that I know and I just stand there … and you’ve got people coming off the trains and they’ve got homes … and you’ve got people living at the backpackers’ places and all.’ (Lynda)

Fights erupted because of queue jumping. Some reported being the victim of or witnessing violence, or being intimidated, and avoided certain CFS to ensure personal safety:

‘I don’t like being in the middle [of a queue] … if you want to be the first well that’s a challenge, because you get the regulars who are the first … And then you get people with their friends and say oh come and stand here, and then you get people behind shouting at them. It’s actually the queue that you have to put up with; it’s not the people that give you the soup.’ (Sandy)

At the first sign of violence mobile food van volunteers pack up and leave. Subsequently, queue conflict prompted de-escalating behaviours by adjacent others to avoid volunteers leaving and everyone going hungry:

‘You just line up and as long as you’re acting in good behaviour you get fed but if you’re a little bit fighting or shouting or screaming … if they want to use the services they’ve got to be quiet.’ (Nathan)

Recipients wanted ‘less pampering’, more independence and social connection when it comes to food. They wanted autonomy to choose their own food, as Lynda, who had diabetes, explained:

‘Okay, so choice, having some control on what you get to put on your plate … Then they know you are getting fed how you want to get fed … You got diabetics, not only me, they do line up for feeds also … let them choose what they want to eat.’ (Lynda)

A personalised connection with service providers was desired rather than just ‘being fed’ in a queue:

‘“How are you this morning Sandy?” or something like this, instead of just handing it [the food] out. “Do you want a pie or do you want some water?” … you know, treating you like ordinary [person]. I think that’s lacking there. That’s what they do in prison you know.’ (Sandy)

Recipients wanted choice in hostels and in food parcels. The $AU 20 supermarket food voucher provided by some CFS was seen as preferable because it enabled recipients to purchase food of their choice. Additional soup vans and volunteers were called for, with assurances that mobile services would carry sufficient food on board to allay fears of missing out. More reliable and inviting places to eat, preferably without the need to queue, were recommended; along with awards to acknowledge CFS volunteers.

### Food memories and inclusion

The theme of ‘food memories and inclusion’ reflected a desire for commensality and reinstating food as a shared social activity. Long-term recipients spoke of a deeper meaning of food and recounted memories of nostalgic eating occasions. Barbeque meals and roast dinners were a rare luxury and in stark contrast to their current CFS fare, namely soup served in disposable cups, meat pies, sandwiches or meals eaten from plastic containers while standing in the park. Recipients were keen to participate in normal societal eating. The importance of a ‘proper dinner’, ‘an evening meal’, ‘proper meat [for barbecues] and salad’ and a ‘decent Sunday meal’ was discussed.

Recipients felt CFS did not want them to miss out on celebratory foods such as Easter eggs or Christmas fare and knew donations of these foods peaked at this time:

‘They do have a lot of snack food here especially after Christmas. I can’t afford to get sick at the moment, so like it’s nice but don’t do it to yourself … It’s so easy to eat comfort food and it’s great when you need comforting … but I’ve been feeling a bit sick so that could be my diabetes.’ (Jane)

Recipients wanted normalised or culturally appropriate eating occasions such as barbecues or Sunday dinners. These were seen as homely and offering comfort as well as opportunities for socialisation and commensality:

‘Give them something decent for the weekends, a Sunday roast or something, they’d love to look forward to a decent meal on a Sunday … and their homemade desserts on top of that, whatever, give them the luxury for themselves.’ (Lynda)

## Discussion

The current research found that CFS recipients in Perth are acutely aware of the shortcomings of CFS and have specific recommendations for improvements.

Recipients were grateful for the food but were resigned to limited variety and poor quality. They were cognisant of CFS limitations and reluctant to criticise. Despite this, the monotony of food was especially salient for long-term users. Monotony, quality and access issues, as well as the significant time and effort required to access three meals per day, are consistent with previous findings in Vancouver^(^ ^7^ ^)^, Quebec^(^ ^24^ ^)^ and Melbourne^(^ ^5^ ^)^ and are a consequence of *ad hoc* donations of unsaleable food.

Similar to the present study, CFS users in Melbourne voiced concerns about the monotony, quality and variety of food, the indignity of queuing, the fear of missing out and the desire to socialise and relax over food^(^ ^19^ ^)^. Interviewees in that study also recommended providing opportunities to socialise and relax in a dignified manner at services, as well as coordination and collaboration across CFS.

Recipients wanted more healthy, high-quality and varied food, especially those with chronic health conditions. For long-term CFS recipients, a diet heavily reliant on less nutritious foods may exacerbate existing conditions such as diabetes^(^ ^8^ ^,^ ^25^ ^)^ or induce the development of chronic disease.

In the USA, ‘nutrition-focused’ procurement policies restrict the procurement of unhealthy food within food banks^(^ ^26^ ^)^. Given the prevalence of obesity and poverty^(^ ^27^ ^)^, it is both important and urgent that policies such as the New York Food Bank’s ‘No Soda, No Candy’ policy are considered in Australian CFS^(^ ^28^ ^)^. Concerns that corporate food donors may be unsupportive of nutrition-focused policies or that relationships may be compromised have been unfounded in the USA^(^ ^29^ ^,^ ^30^ ^)^. Australian CFS’ reliance on unsaleable waste food from donors rather than purchased food may be a limiting factor in the implementation of nutrition-focused food procurement. Australian supermarkets hold the power in the food system and all have made corporate social responsibility statements relating to improving the health of the community^(^ ^31^ ^)^. They could strengthen their position from one of diverting food waste to charity to providing a consistent and reliable supply of nutritious food that supports nutrition-focused food procurement.

The considerable effort and work involved in finding CFS and securing food, akin to ‘a full-time job’, is consistent with the experience of CFS recipients Vancouver, Canada. Described as doing ‘the rounds’, people moved to various CFS for meals, for example breakfast at a soup kitchen, lunch at a charity and dinner at another church group^(^ ^7^ ^)^. The reliance on walking to access CFS in the present study is consistent with other findings^(^ ^32^ ^)^; however, walking in temperatures of over 40°C is a concern in Perth, particularly for older people or young children. Foot problems are common among homeless people due to inadequate footwear, extended walking and standing, and poor hygiene^(^ ^33^ ^)^. Use of free air-conditioned ‘city loop’ buses provided some respite. Some recipients incurred fines as they could not afford public transport, increasing their debt, anxiety and sense of hopelessness.

An unavoidable consequence of the work involved in securing food assistance is that it reduces the time available for dealing with housing, health and legal matters. Promising international models such as social supermarkets^(^ ^34^ ^)^ or Fresh Place^(^ ^35^ ^)^ offer a range of services such as employment assistance in addition to food, providing pathways to food security.

Information access was challenging, with information sharing among recipients important. Recipients recommended a website be developed. A recent Australian Government mobile phone application aiming to connect homeless people to health, housing and well-being services did not give specific locations or times for accessing CFS in Perth^(^ ^36^ ^)^. Accurate digital information on CFS via mobile offers promise as homeless Australians have higher rates of smartphone ownership than the general population^(^ ^37^ ^)^.

Publicly queuing for food was a salient issue, symbolising social exclusion from mainstream society and inequality^(^ ^38^ ^)^. Queues were associated with a loss of dignity, fears of violence and missing out on food. The shame and the indignity of queuing for food have been reported in Dutch food banks^(^ ^39^ ^)^. Similar to Salonen, queues in the present study reflect mutual surveillance with some recipients keenly observing others and questioning their eligibility or worthiness for food charity. In-queue friction was de-escalated by those nearby and is consistent with queues as symbolising an embryonic social system with a set of norms for controlling conflict^(^ ^40^ ^)^. In contrast, Finnish ‘breadlines’ queues are described as functioning social spaces for new social connections and community building^(^ ^41^ ^)^.

Recipients sought improvements that increase individual agency and control. For example, being able to choose and cook food regains a sense of power and independence, while ‘being fed’ erodes dignity^(^ ^42^ ^)^. Suggestions of more recipient-centred approaches, such as a ‘proper dinner’, imply seated meal services which foster commensality. The importance of collective eating should not be discounted, as Fischler argues: ‘Eating together is seen as bringing people together … it means building or rebuilding a common destiny’^(^ ^43^ ^)^ (p. 20).

A small number of Australian programmes such as Café Meals^(^ ^44^ ^)^ and Social Spoons^(^ ^44^ ^)^ provide opportunities for socialisation. Apart from The Salvation Army’s seated Christmas dinner in Perth, there is scant information on other CFS providing regular ‘proper’ meals. Social inclusion underpins some international examples, including the Caritas homeless services (Spain)^(^ ^45^ ^)^ and Lobby Restaurants (Germany) which offers a three-course meal to both income and non-income earners with a tiered price structure^(^ ^46^ ^)^. Restaurant staff include formerly homeless people, welfare recipients and the unemployed^(^ ^46^ ^)^. These examples address the three vectors of exclusion, namely poverty, isolation and a lack of life orientation. Each dining room has a waiting room where people can sit or read the newspaper before or after the meal. Other services include showers, laundry and health care. The result is a comprehensive and supportive environment that facilitates social engagement and ‘normalises’ eating^(^ ^45^ ^)^.

Charitable food provision is more than parcels; food is deeply symbolic and has a social context^(^ ^47^ ^)^. Recipients recalled food memories and yearned for a ‘decent Sunday meal’. These calls echo seminal UK work investigating the cultural and social significance of a cooked dinner^(^ ^48^ ^)^. A cooked dinner consisting of meat (usually a roast joint), potatoes and a least one additional vegetable plus gravy constitutes a ‘proper’ meal. Sausages and cheaper meat cuts were not considered ‘proper’ yet were eaten during the week to save money and to allow for a ‘proper dinner’ on Sunday. These comments call for improved food quality and commensality and are consistent with other recommendations.

From a broader perspective, recipients’ views on the CFS in inner-city Perth describe what constitutes voluntary failure of the system, where government has largely abdicated its role in funding and setting policy for food for people in need. The Australian charitable food system (including foodbanks) has limited, if any, government support and is an ‘emergency intervention’. It does not meet long-term need due to limited occasions of use^(^ ^6^ ^,5,^ ^49^ ^)^ and has been referred to as a ‘voluntary failure’ due to the significant unmet need^(^ ^10^ ^)^. The current findings provide evidence of two of the four failures described in Salamon’s Theory of Voluntary Failure: philanthropic insufficiency and philanthropic paternalism^(^ ^10^ ^)^.

First, according to recipients, the CFS fails to deliver the ‘public good’ (of providing food to those in need) due to philanthropic insufficiency, simply not having enough resources to meet demand. Our recipients acknowledged that CFS were experiencing resource pressure with increasing demand and tried to help mitigate this by moving between CFS and not relying solely on one service. Other indicators of unmet demand included limited food variety, poor quality, insufficient food and operational limitations, reflected in descriptions of dated brochures, difficultly seeking assessment appointments and the considerable burden in accessing food.

Second, the findings demonstrate philanthropic paternalism where donors and volunteers work with people in ways that undermine their dignity and autonomy. Dignity erodes when private activities such as eating and sleeping are conducted in full public view^(^ ^50^ ^)^. Having to queue for food in parks or retrieve food from rubbish bins exacerbates this. Increasing control and surveillance over the use of public spaces for private activities has a detrimental impact on the lives of homeless people, when queuing for food or having to resort to eating discarded food from rubbish bins.

Wills’ Melbourne study also found evidence of the same two voluntary failures and concluded that innovative solutions were required to overcome increasing demands on food banking resources given the structural and pervasive nature of the problem and recommended more dignifying means of ensuring food security^(^ ^6^ ^)^.

Given that food security can be understood as a public good that is not adequately provided for by government or by for-profit businesses, three market failures apply. Voluntary failure sits adjacent to market failure and government failure which is due to inadequate support for under- or unemployment, an insufficient welfare safety net, and an absence of government policy and funding for food assistance programmes. Implementing recipients’ recommendations for improvement within existing CFS models may be challenging, particularly when the broader landscape of failures is considered. In short, the current system may lack the flexibility to respond to recipient recommendations.

### Strengths, limitations and future research

The present study is the first Western Australian one gathering CFS recipients’ perspectives and their recommendations for improvement. The sample size is small and located in inner-city Perth, so results may not be generalisable. Further research targeting regional and remote areas in Western Australia is recommended as experiences and recommendations may differ.

We recruited and heard the perspectives of long-term CFS recipients using in-depth interview methodology, which elicits rich contextual data often not reported. The lived experience of this unique sample identified systemic problems with charitable food provision that may not have been captured by other methods. Further research with a larger sample is needed to quantify the extent of the issues. Exploring the perspectives of CFS managers is also advisable.

Based on the findings, alternative models to CFS provision that focus on the recipients’ needs should be assessed, trialled and evaluated. The issues identified call for a ‘systems approach’ to research on hunger relief logistics and supply chain issues^(^ ^51^ ^)^. Applying Salamon’s Theory of Voluntary Failure to the recipient experience highlighted broader challenges facing the system and locates them in a wider political and policy context.

## Conclusion

Although grateful, long-term recipients of CFS described what constitutes philanthropic and paternalistic voluntary failures. Their service improvement recommendations can help meet their nutritional and social needs. A successful system prioritises nutritious, good-quality food and individual need, and promotes dignity and social inclusion. The nature of Australian CFS may limit the realisation of recipients’ recommendations.
